# Widespread Dissemination of Plasmid-Mediated Tigecycline Resistance Gene *tet*(X4) in *Enterobacterales* of Porcine Origin

**DOI:** 10.1128/spectrum.01615-22

**Published:** 2022-09-20

**Authors:** Qin Wang, Changwei Lei, Hansen Cheng, Xue Yang, Zheren Huang, Xuan Chen, Zijing Ju, Haoyu Zhang, Hongning Wang

**Affiliations:** a Key Laboratory of Bio-Resource and Eco-Environment of Ministry of Education, College of Life Sciences, Sichuan University, Chengdu, Sichuan Province, People’s Republic of China; b Animal Disease Prevention and Food Safety Key Laboratory of Sichuan Province, Chengdu, People’s Republic of China; China Agricultural University

**Keywords:** *Enterobacterales*, tigecycline resistance, *tet*(X4), evolutionary relationship, genetic environments

## Abstract

The emergence of the plasmid-mediated high levels of the tigecycline resistance gene has drawn worldwide attention and has posed a major threat to public health. In this study, we investigated the prevalence of the *tet*(X4)-positive *Enterobacterales* isolates collected from a pig slaughterhouse and farms. A total of 101 tigecycline resistance strains were isolated from 353 samples via a medium with tigecycline, of which 33 carried *tet*(X4) (9.35%, 33/353) and 2 carried *tet*(X6) (0.57%, 2/353). These strains belong to seven different species, with Escherichia coli being the main host bacteria. Importantly, this report is the first one to demonstrate that *tet*(X4) was observed in Morganella morganii. Whole-genome sequencing results revealed that *tet*(X4)-positive bacteria can coexist with other resistance genes, such as *bla*_NDM-1_ and *cfr*. Additionally, we were the first to report that *tet*(X4) and *bla*_NDM-1_ coexist in a Klebsiella quasipneumoniae strain. The phylogenetic tree of 533 *tet*(X4)-positive E. coli strains was constructed using 509 strains from the NCBI genome assembly database and 24 strains from this study, which arose from 8 sources and belonged to 135 sequence types (STs) worldwide. We used Nanopore sequencing to interpret the selected 21 nonclonal and representative strains and observed that 19 *tet*(X4)-harboring plasmids were classified into 8 replicon types, and 2 *tet*(X6) genes were located on integrating conjugative elements. A total of 68.42% of plasmids carrying *tet*(X4) were transferred successfully with a conjugation frequency of 10^−2^ to 10^−7^. These findings highlight that diverse plasmids drive the widespread dissemination of the tigecycline resistance gene *tet*(X4) in *Enterobacterales* of porcine origin.

**IMPORTANCE** Tigecycline is considered to be the last resort of defense against diseases caused by broad-spectrum resistant Gram-negative bacteria. In this study, we systematically analyzed the prevalence and genetic environments of the resistance gene *tet*(X4) in a pig slaughterhouse and farms and the evolutionary relationship of 533 *tet*(X4)-positive Escherichia coli strains, including 509 *tet*(X4)-positive E. coli strains selected from the 27,802 assembled genomes of E. coli from the NCBI between 2002 and 2022. The drug resistance of tigecycline is widely prevalent in pig farms where tetracycline is used as a veterinary drug. This prevalence suggests that pigs are a large reservoir of *tet*(X4) and that *tet*(X4) can spread horizontally through the food chain via mobile genetic elements. Furthermore, tetracycline resistance may drive tigecycline resistance through some mechanisms. Therefore, it is important to monitor tigecycline resistance, develop effective control measures, and focus on tetracycline use in the pig farms.

## INTRODUCTION

In recent years, antibiotic resistance has become a serious concern, posing a major threat to human health and food safety owing to the increased use of human and agricultural antibiotics, which has created selection pressure contributing to resistance (1). Tetracycline antimicrobials are effective against a wide spectrum of pathogens, including Gram-positive and Gram-negative bacteria and atypical organisms (2). Tigecycline is considered a last-resort antibiotic for the treatment of severe infections caused by extensively drug-resistant bacteria (3). However, reports of clinical resistance to tigecycline have increased since 2007, and the initial discovery of tetracycline resistance was largely owing to the expression of different efflux pump genes (*tet*A-E and *tet*L) and ribosome protection protein-encoding genes (*tet*M and *tet*O) (4).

*tet*(X) genes can encode a flavin-dependent monooxygenase that not only catalyzes the efficient degradation of a broad range of tetracycline analogs but also confers resistance to these antibiotics *in vivo*. To date, 47 *tet*(X) variants have been identified (3, 5–13). *tet*(X) genes are widely present in a variety of hosts, such as Escherichia coli, Klebsiella pneumoniae, Proteus mirabilis, Acinetobacter baumannii, and Citrobacter freundii, in humans and animals (14–18). Additionally, *tet*(X) genes occasionally coexist with *bla*_NDM_, *mcr-1*, and *cfr*, which confer resistance to carbapenem, colistin, and oxazolidinone, respectively (19–21). Prior to the discovery of the plasmid-carrying *tet*(X) gene variant, a small number of tigecycline resistance bacteria were reported in 2005 and subsequently discovered, of which most were Gram-negative bacteria (22–26). The rapid spread of the plasmid-mediated *tet*(X) gene variant to tigecycline resistance would complicate the treatment of multidrug-resistant infections and pose a threat to human health. The plasmid-carrying *tet*(X) and its variants are involved in a new mechanism of tigecycline resistance in humans and animals. In 2019, a study revealed the plasmid-mediated tigecycline resistance genes *tet*(X3) and *tet*(X4) in *Enterobacteriaceae* and Acinetobacter in China, posing a major threat to global health (3, 20). Subsequently, plasmids including the IncX1 plasmid and fusion plasmids, such as IncX1-IncFIA-IncFIB-IncFIC, IncFIA-IncHI1B-IncHI1A, IncX1-IncN, and IncY-IncX1-IncFIA-IncFIB, have been reported to mediate the transmission of *tet*(X) and others (27, 28). The appearance of strains, which simultaneously carry super antibiotic resistance genes, and the multiple types of mobile genetic elements, which mediate the spread of resistance genes, can exacerbate the situation of global antibiotic resistance.

Although tigecycline is used in human medicine and is prohibited in veterinary medicine, tetracycline antibiotics are used widely in livestock and poultry breeding in China. In particular, oxytetracycline and aureomycin are used occasionally in pig breeding and may cause cross-drug resistance and the spread of tetracycline resistance genes. However, the prevalence of the plasmid-mediated tigecycline resistance genes in *Enterobacterales* of pig origin in Sichuan Province remains unknown. In this study, we detected the prevalence of *tet*(X4) in a pig slaughterhouse and 10 pig farms and analyzed its genetic environmental diversity in Sichuan Province. Furthermore, we investigated the evolutionary relationship of *tet*(X4)-positive E. coli isolates worldwide. We observed multiple Gram-negative bacteria carrying the *tet*(X4) gene and demonstrated its genetic environmental diversity.

## RESULTS

### Prevalence of *tet*(X4) in a pig slaughterhouse and farms in Sichuan Province.

In total, 101 tigecycline resistance strains were selected, of which 33 carried *tet*(X4) (9.35%, 33/353) and 2 carried *tet*(X6) (0.57%, 2/353). The 33 *tet*(X4)-positive strains were observed predominantly in E. coli (72.72%), followed by K. pneumoniae (12.12%), M. morganii (6.06%), *K. quasipneumoniae* (3.03%), Proteus vulgaris (3.03%), and P. mirabilis (3.03%). The remaining two *tet*(X6)-positive strains belonged to Proteus terrae subsp. *cibarius*. To the best of our knowledge, we are the first to report that *tet*(X4) is observed in M. morganii. The strains isolated from the slaughterhouse belonged to six species, and the strains isolated from the pig farms belonged to four species. Besides, the monoclonal strains were from different samples (Table 1).

**TABLE 1:** Information on 35 *tet*(X4)/*tet*(X6)-carrying strains

Strain	Source^*a*^	Species	ST	MIC of tigecycline (mg/L)	Resistance phenotype^*b*^	Genome accession
JZ30	S	P. vulgaris		>256	TGC, DOX, FFC, PMB, SXT, FOX, GEN, TET, CAZ, AMC, CRO, ATM	JALMET000000000
JZ35	S	P. terrae subsp. *cibarius*		32	TGC, DOX, FFC, PMB, SXT, GEN, TET, AMC, CRO, AMK	JALMES000000000
JZ49	S	P. mirabilis		>256	TGC, DOX, FFC, PMB, SXT, GEN, TET, CAZ, AMC, CRO, ATM	JALMER000000000
JZ47	S	K. pneumoniae	ST629	32	TGC, DOX, FFC, SXT, FOX, FOS, GEN, TET, CAZ, AMC, CRO, ATM	JALMEQ000000000
JZ2	S	*K. quasipneumoniae*		128	TGC, DOX, FFC, MEM, SXT, FOX, FOS, TET, CAZ, AMC, CRO, ATM	JALMEP000000000
JZ18	S	K. pneumoniae	ST25	128	TGC, DOX, FFC, SXT, FOS, GEN, TET, CAZ, CRO, AMK, ATM	JALMEO000000000
JZ19	S	E. coli	ST793	32	TGC, DOX, FFC, SXT, GEN, TET, CRO, AMK, ATM	JALMEN000000000
JZ50	S	E. coli	ST195	64	TGC, DOX, FFC, SXT, TET, CAZ, CRO, ATM	JALMEM000000000
JZ21	S	E. coli	ST48	32	TGC, DOX, FFC, SXT, GEN, TET, CAZ, CRO, ATM	JALMEL000000000
JZ27	S	E. coli	ST10671	32	TGC, DOX, FFC, GEN, TET, CRO, AMK, ATM	JALMEK000000000
XY36	F1	M. morganii		256	TGC, DOX, FFC, PMB, SXT, FOS, TET	JALMEJ000000000
XY10	F1	E. coli	ST101	32	TGC, DOX, FFC, SXT, TET	JALMEI000000000
XY3	F1	E. coli	ST101	16	TGC, DOX, FFC, CIP, SXT, TET	JALMEH000000000
XY1	F1	E. coli	ST218	16	TGC, DOX, FFC, SXT, GEN, TET	JALMEG000000000
XY7	F1	E. coli	ST1684	32	TGC, DOX, FFC, SXT, TET, ATM	JALMEF000000000
XY14	F1	E. coli	ST195	64	TGC, DOX, FFC, SXT, GEN, TET, CAZ, CRO	JALMEE000000000
DM22	F4	E. coli	ST195	64	TGC, DOX, FFC, SXT, GEN, TET, CAZ, CRO	JALMED000000000
DM3	F4	E. coli	ST195	32	TGC, DOX, FFC, SXT, TET, CAZ, CRO	JALMEC000000000
DM13	F4	E. coli	ST8076	16	TGC, DOX, FFC, SXT, TET, AMC	JALMEA000000000
TQ3	F5	E. coli	ST195	64	TGC, DOX, FFC, SXT, GEN, TET, CAZ, CRO	JALMDZ000000000
TQ5	F5	E. coli	ST10	16	TGC, DOX, FFC, SXT, TET	JALMDY000000000
TQ6	F5	E. coli	ST761	64	TGC, DOX, FFC, TET, CAZ, CRO	JALMDX000000000
TQ2	F5	E. coli	ST10	16	TGC, DOX, FFC, SXT, TET, AMC	JALMDW000000000
TQ13	F5	E. coli	ST877	32	TGC, DOX, CIP, SXT, GEN, NOR, TET	JALMDV000000000
TQ41	F5	E. coli	ST69	32	TGC, DOX, FFC, GEN, TET, AMK	JALMDU000000000
TQ25	F5	E. coli	ST12984	16	TGC, DOX, FFC, CIP, SXT, GEN, NOR, TET, CAZ, CRO, ATM	JALMDT000000000
TQ55	F5	E. coli	ST48	16	TGC, DOX, FFC, SXT, TET	JALMDS000000000
TQ17	F5	E. coli	ST165	32	TGC, DOX, FFC, TET	JALMDR000000000
TQ30	F5	K. pneumoniae	ST25	64	TGC, DOX, FFC, CIP, SXT, FOS, TET	JALMDQ000000000
TQ11	F5	K. pneumoniae	ST35	128	TGC, DOX, FFC, FOS, TET	JALMDP000000000
TQ12	F5	P. terrae subsp. *cibarius*		128	TGC, DOX, FFC, SXT, TET, AMC	JALMDO000000000
TQ28	F5	M. morganii		128	TGC, DOX, FFC, CIP, SXT, GEN, TET	JALMDN000000000
DW10	F8	E. coli	ST46	128	TGC, DOX, FFC, TET	JALMDM000000000
DW28	F8	E. coli	ST617	64	TGC, DOX, FFC, CIP, SXT, NOR, TET	JALMDL000000000

a S, pig slaughterhouse; F, pig farms.

b TGC, tigecycline; TET, tetracycline.

### Antimicrobial susceptibility testing.

Antimicrobial susceptibility testing using 17 antimicrobial agents revealed that these positive strains widely exhibited a multidrug resistance (MDR) phenotype. All tigecycline resistance strains were resistant to tetracycline and doxycycline (DOX), and more than 90% of the strains were resistant to florfenicol (FFC). A total of 24 E. coli strains carrying *tet*(X4) were resistant to tetracycline (100%; *n* = 24), DOX (100%; *n* = 24), FFC (95.83%; *n* = 23), trimethoprim-sulfamethoxazole (SXT; 79.17%; *n* = 19), ceftriaxone sodium (CRO; 45.83%; *n* = 11), gentamicin (GEN; 41.67%; *n* = 10), ceftazidime (CAZ; 37.50%; *n* = 9), aztreonam (ATM; 25%; *n* = 6), ciprofloxacin (CIP; 20.83%; *n* = 5), amikacin (AMK; 12.50%; *n* = 3), norfloxacin (NOR; 12.50%; *n* = 3), and amoxicillin (AMC; 22.86%; *n* = 8). All E. coli isolates were susceptible to polymyxin B (PMB), cefoxitin (FOX), meropenem (MEM), and fosfomycin (FOS). The lowest MIC of tigecycline was 16 mg/L; however, strangely, the highest MICs of tigecycline were greater than 256 mg/L, including P. vulgaris and P. mirabilis (Table 1).

### Diversity of sequence types and resistance genes.

The 35 isolates were analyzed using whole-genome sequencing and multilocus sequence type (MLST) analysis, which revealed that all the 24 E. coli isolates had a variety of STs (*n* = 16), including ST195 (*n* = 6), ST48 (*n* = 2), ST101 (*n* = 2), ST10 (*n* = 2), ST793 (*n* = 1), ST10671 (*n* = 1), ST218 (*n* = 1), ST1684 (*n* = 1), ST8074 (*n* = 1), ST761 (*n* = 1), ST877 (*n* = 1), ST69 (*n* = 1), ST165 (*n* = 1), ST46 (*n* = 1), ST617 (*n* = 1), and a new ST, ST12984. Three different STs, including ST25 (*n* = 2), ST629 (*n* = 1), and ST35 (*n* = 1), were observed in four K. pneumoniae isolates. All strains carried at least one aminoglycoside resistance gene, such as *aac*, *aph*, and *aadA*. Additionally, various kinds of β-lactam resistance genes were observed, such as *bla*_CTX-M-14_ (*n* = 7, 20%), *bla*_CTX-M-55_ (*n* = 1, 2.86%), *bla*_DHA-1_ (*n* = 5, 14.29%), *bla*_CMY-2_ (*n* = 2, 5.71%), *bla*_CMY-38_ (*n* = 2, 5.71%), *bla*_NDM-1_ (*n* = 1, 2.86%), and *bla*_OXA-1_ (*n* = 1, 2.86%). Furthermore, the phenicols (*floR*; 35/35), sulfonamides (*sul*; 30/35), trimethoprims (*dfrA*; 28/35), quinolones (*qnrS*; 26/35), and lincosamide [*lnu*(F); 21/35] resistance genes were also detected. A strain of P. terrae subsp. *cibarius* carrying both multiple resistance gene *cfr* and tigecycline resistance gene *tet*(X6) was observed (see Table S1 in the supplemental material). In addition, the remaining 66 tigecycline resistance strains, including P. mirabilis (*n* = 52), P. vulgaris (*n* = 1), K. pneumoniae (*n* = 7), E. coli (*n* = 4), and Enterobacter hormaechei (*n* = 2) without *tet*(X4) or *tet*(X6) had some other tetracycline resistance genes like *tet*(A), *tet*(B), *tet*(D), *tet*(H), *tet*(J), and *tet*(M). Furthermore, 3 P. mirabilis strains from a pig slaughterhouse had tigecycline resistance gene cluster *tmexCD3-toprJ3*.

### Phylogenomic analysis.

**FIG 1:**
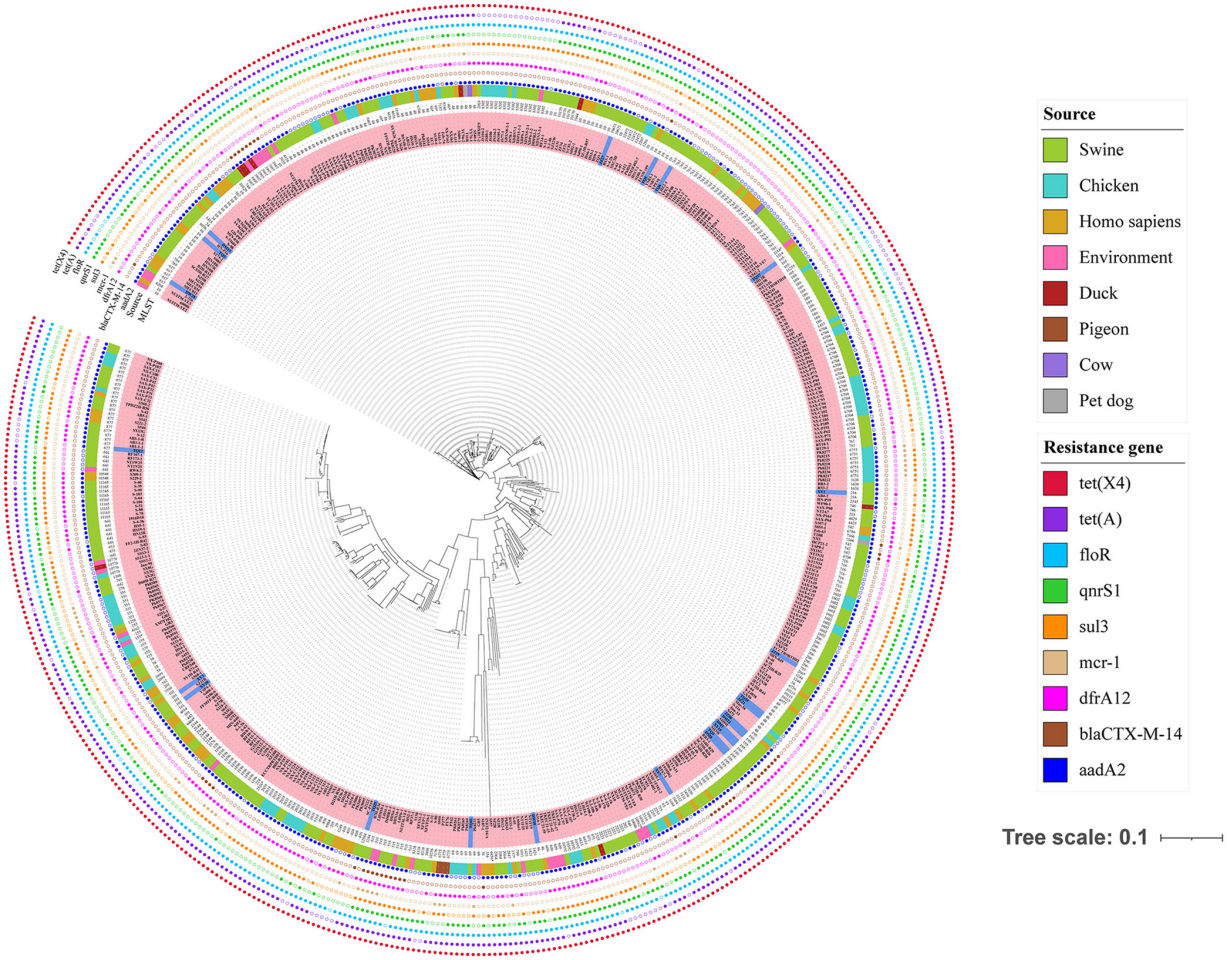
Phylogenetic tree of 533 *tet*(X4)-positive E. coli isolates, including 24 E. coli isolates in this study and 509 E. coli isolates from NCBI genome assembly database. Blue represents the E. coli in this study and pink represents the E. coli screened from NCBI. The source of strains, MLST, and some resistance genes were illustrated.

**FIG 2:**
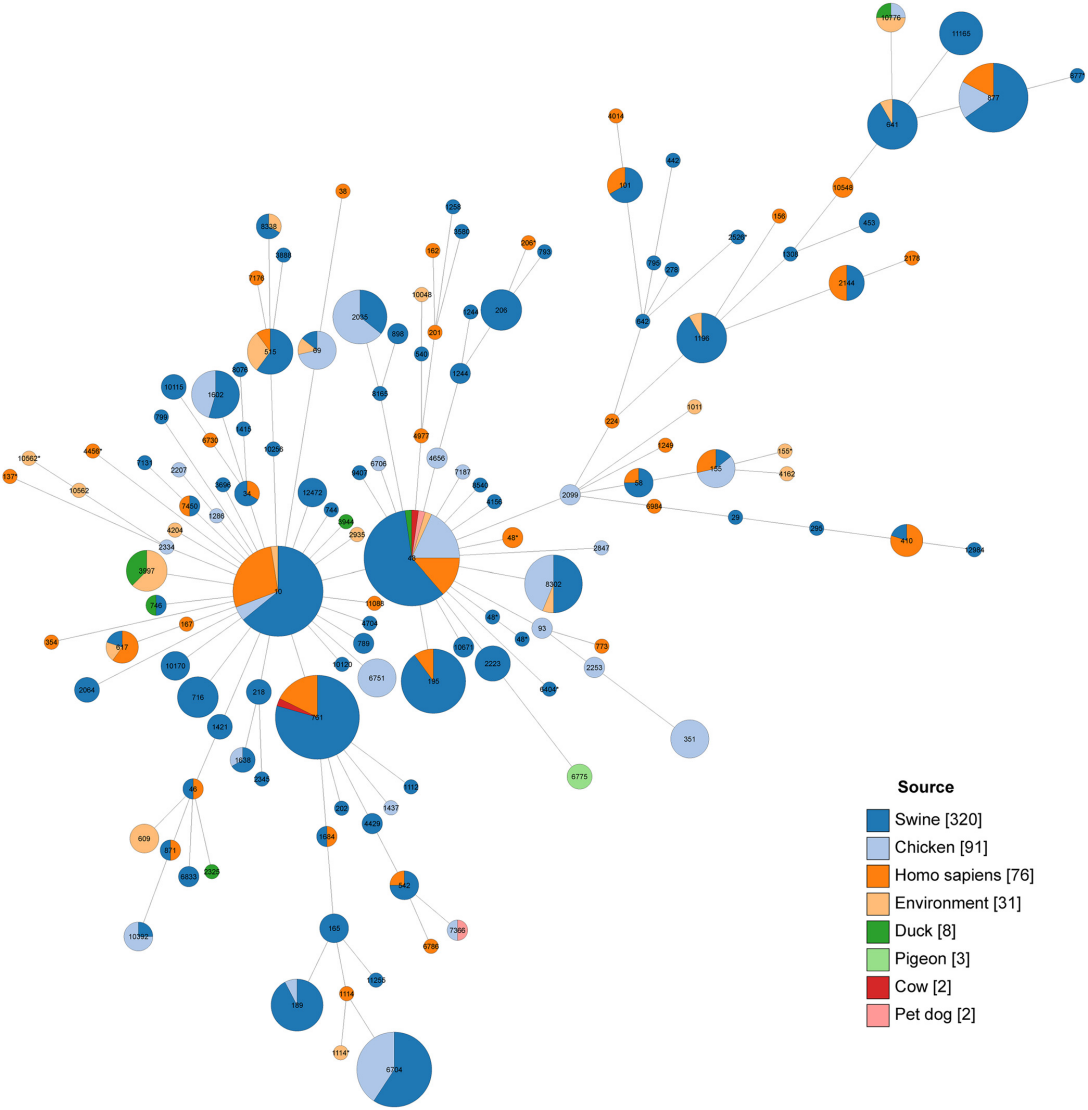
Core-genome MLST allelic profiles of E. coli, including 24 *tet*(X4)-positive E. coli isolates in this study and 509 *tet*(X4)-positive E. coli isolates from the NCBI genome assembly database.

Based on the stand-alone BLAST, a total of 509 *tet*(X4)-positive E. coli strains were observed in the 27,802 assembled genomes of E. coli from NCBI between 2002 and 2022. These strains were derived from pigs, humans, chickens, the environment, ducks, pigeons, cows, and pet dogs around the world, and the pig is the most important source of the *tet*(X4) gene (see Table S2 in the supplemental material). Based on single nucleotide polymorphisms (SNPs) of core genomes, the phylogenetic tree of 533 *tet*(X4)-positive E. coli strains, including 24 strains in this study, was constructed. The 533 strains belonged to 135 different ST types, and ST48, ST10, and ST761 were the main ST types. The SNP range of 533 E. coli strains ranged from 0 to 44,532, and there were 304 distinct clones, 66 of which were clonally transmitted between people, animals, and the environment, and they belonged to 45 STs, with ST761, ST6704, and ST48 being the primary STs. The SNP range of the 533 E. coli strains varied from 0 to 44,532. Besides, more than 99.81% of strains coharbored *floR* and *tet*(X4), and more than 87.42% of strains coharbored *tet*(A) and *tet*(X4) (see Table S3 in the supplemental material; Fig. 1 and 2).

### Genetic diversity and diverse plasmids carrying *tet*(X4).

By Nanopore sequencing, the complete structural sequence of mobile genetic elements carrying *tet*(X4)/*tet*(X6) was obtained from 21 nonclonal and representative strains. In total, 19 *tet*(X4)-harboring plasmids were classified into 8 replicon types, and 2 *tet*(X6) genes were located on integrative and conjugative elements (ICEs). The IncX1 (*n* = 9) type of plasmids was observed to be the most prevalent, and their lengths ranged between 31,271 bp and 73,084 bp. These plasmids were found in several species as well as various pig farms and slaughterhouses, demonstrating the global dissemination of plasmids of the IncX1 type. Additionally, there were some plasmid types that have been reported in previous studies, such as IncFIA-IncHI1A-IncHI1B (*n* = 3), IncC (*n* = 2), IncFIB-IncFIC (*n* = 1), IncFIA-IncFIB-IncX1 (*n* = 1), IncFIA-IncHI1A-IncHI1B-Col (*n* = 1), and IncFIA-IncHI1A-IncHI1B-IncX1 (*n* = 1). However, the heterozygous plasmid containing IncX3 of IncFIA-IncHI1A-IncHI1B-IncX3 (*n* = 1) was first reported in this study (Table 2). Importantly, more than 99% similarity existed in pXY14-*tet*(X4), pXY10-*tet*(X4), pJZ2-*tet*(X4), pTQ3-*tet*(X4), pJZ50-*tet*(X4), pJZ18-*tet*(X4), and pJZ19-*tet*(X4). However, these plasmids were divided from nonclone strains. The *tet*(X4) gene in M. morganii is located on a heterozygous plasmid, IncFIA-IncHI1A-IncHI1B. Remarkably, *tet*(X4) and *cfr*/*bla*_NDM-1_ were observed in the same strain, and this is the first study to report that *tet*(X4) and *bla*_NDM-1_ coexisted in a *K. quasipneumoniae* strain. *tet*(X4) and *bla*_NDM-1_ are located on plasmids IncX1 and IncX3, respectively. Furthermore, the coverage rate and the similarity between ICEPmiChn-JZ35 and ICEPmiChn-TQ12 were 75% and 99.55%, respectively. Compared with the first reported ICE SXT/R391 carrying *tet*(X6), ICEPmiChn-JZ35 had an 82% coverage rate and 96.93% similarity, whereas ICEPmiChn-TQ12 had a 64% coverage rate and 97.20% similarity.

**TABLE 2:** The results of 21 *tet*(X4)/*tet*(X6)-carrying strains by long-read Nanopore sequencing

Strain	Location of *tet*(X)	Length (bp)	MGE^*a*^ type	Resistance genes on *tet*(X)-harboring MGE	Conjugation frequency	Accession no.
JZ30	pJZ30-*tet*(X4)	185,363	IncA/C2	*tet*(X4), *sul1*, *aac(3)-VIa*, *aadA1*, *erm(42)*, *bla*_CMY-2_, *qacE*, *floR*	10^−3^	ON390809
JZ35	ICEPmiChn-JZ35	142,200	ICE	*tet*(X6), *aph(3′)-VIa*, *aadA2b*, *aadA2*, *aph(6)-Id*, *aph(3′')-Ib*, *sul1*, *sul2*, *dfrA19*, *floR*, *qacE*	10^−5^	ON390821
JZ49	pJZ49-*tet*(X4)	197,640	IncA/C2	*tet*(X4), *aac(3)-VIa*, *aadA1*, *erm(42)*, *bla*_CMY-2_, *sul1*, *floR*, *qacE*	10^−5^	ON390810
JZ2	pJZ2-*tet*(X4)	31,272	IncX1	*tet*(X4), *aadA2*, *floR*, *lnu*(F), *tet*(A)	10^−5^	ON390804
JZ18	pJZ18-*tet*(X4)	57,105	IncX1	*tet*(X4), *aadA2*, *bla*_SHV-12_, *floR*, *lnu*(F), *tet*(A)	10^−2^	ON390805
JZ19	pJZ19-*tet*(X4)	57,105	IncX1	*tet*(X4), *aadA2*, *bla*_SHV-12_, *floR*, *lnu*(F), *tet*(A)		ON390806
JZ50	pJZ50-*tet*(X4)	31,250	IncX1	*tet*(X4), *aadA2*, *floR*, *lnu*(F), *tet*(A)	10^−3^	ON390811
JZ21	pJZ21-*tet*(X4)	73,084	IncX1	*tet*(X4), *aph(6)-Id*, *bla*_TEM-1B_, *sul3*, *dfrA14*, *floR*, *mef*(B), *qnrS1*, *formA*, *tet*(A)		ON390807
JZ27	pJZ27-*tet*(X4)	159,632	IncFIB, IncFIC	*tet*(X4), *aph(3′)-Ia*, *aadA1*, *aadA2b*, *erm(42)*, *sul3*, *floR*, *lnu*(F), *qnrS1*, *tet*(A)	10^−4^	ON390808
XY36	pXY36-*tet*(X4)	190,661	IncFIA, IncHI1A, IncHI1B	*tet*(X4), *aadA22*, *bla*_TEM-1B_, *qnrS1*, *floR*, *lnu*(G)		ON390820
XY10	pXY10-*tet*(X4)	31,272	IncX1	*tet*(X4), *aadA2*, *floR*, *lnu*(F), *tet*(A)	10^−6^	ON390818
XY14	pXY14-*tet*(X4)	31,272	IncX1	*tet*(X4), *aadA2*, *floR*, *lnu*(F), *tet*(A)	10^−5^	ON390819
DM13	pDM13-*tet*(X4)	241,960	IncFIA, IncHI1A, IncHI1B, IncX1	*tet*(X4), *aph(3′)-Ia*, *aadA22*, *bla*_TEM-1B_, *bla*_TEM-176_, *dfrA14*, *floR*, *lnu*(G), *qnrS1*, *tet*(A)	10^−7^	ON390802
TQ3	pTQ3-*tet*(X4)	31,271	IncX1	*tet*(X4), *aadA2*, *floR*, *lnu*(F), *tet*(A)		ON390813
TQ6	pTQ6-*tet*(X4)	201,404	IncFIA, IncHI1A, IncHI1B	*tet*(X4), *bla*_TEM-1B_, *floR*, *lnu*(G), *aadA22*, *qnrS1*	10^−4^	ON390814
TQ2	pTQ2-*tet*(X4)	198,381	Col, IncFIA, IncHI1A, IncHI1B	*tet*(X4), *bla*_TEM-1B_, *floR*, *lnu*(G), *aadA22*, *qnrS1*		ON390812
TQ17	pTQ17-*tet*(X4)	90,608	IncFIA, IncFIB, IncX1	*tet*(X4), *aadA2*, *bla*_TEM-1A_, *floR*, *lnu*(F), *qnrS1*, *tet*(A)		ON390815
TQ30	pTQ30-*tet*(X4)	225,359	IncX3, IncFIA, IncHI1A, IncHI1B	*tet*(X4), *bla*_TEM-1B_, *floR*, *lnu*(G), *aadA22*, *qnrS1*	10^−3^	ON390817
TQ12	ICEPmiChn-TQ12	147,546	ICE	*tet*(X6), *aadA2*, *aph(3′)-Ia*, *aph(3′')-Ib*, *aph(6)-Id*, *sul2*, *floR*, *tet*(A)	10^−4^	ON390822
TQ28	pTQ28-*tet*(X4)	187,191	IncFIA, IncHI1A, IncHI1B	*tet*(X4), *bla*_TEM-1B_, *floR*, *aadA1*, *qnrS1*, *lnu*(G)	10^−6^	ON390816
DW28	pDW28-*tet*(X4)	46,856	IncX1	*tet*(X4), *floR*, *lnu*(F), *aadA2*, *tet*(A)	10^−5^	ON390803

a MGE, mobile genetic element.

**FIG 3:**
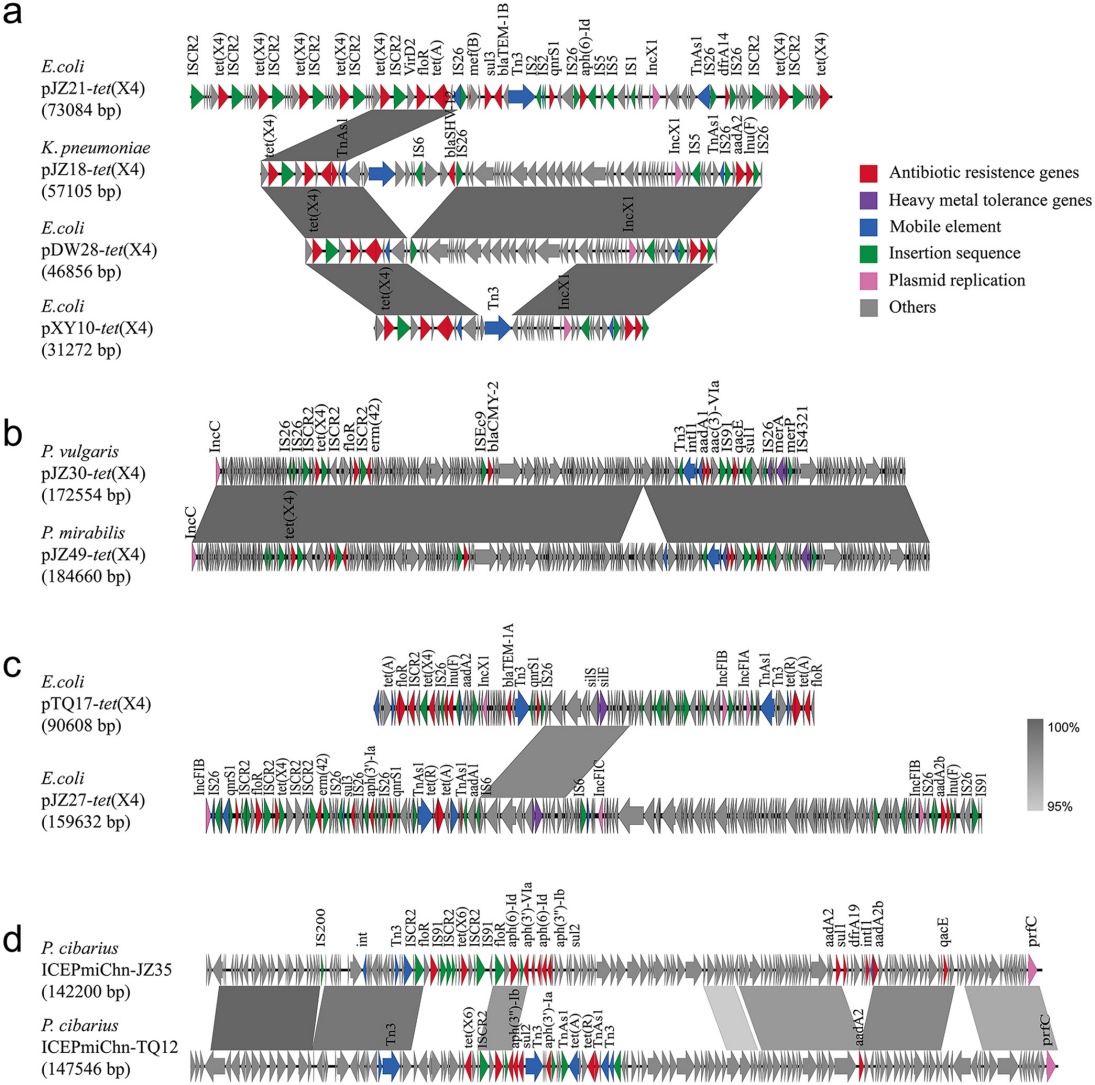
Comparative analysis of the *tet*(X4)-carrying plasmids and *tet*(X6)-bearing ICEs. Comparative analysis of regions of >95% nucleotide sequence homology is marked by gray shading.

**FIG 4:**
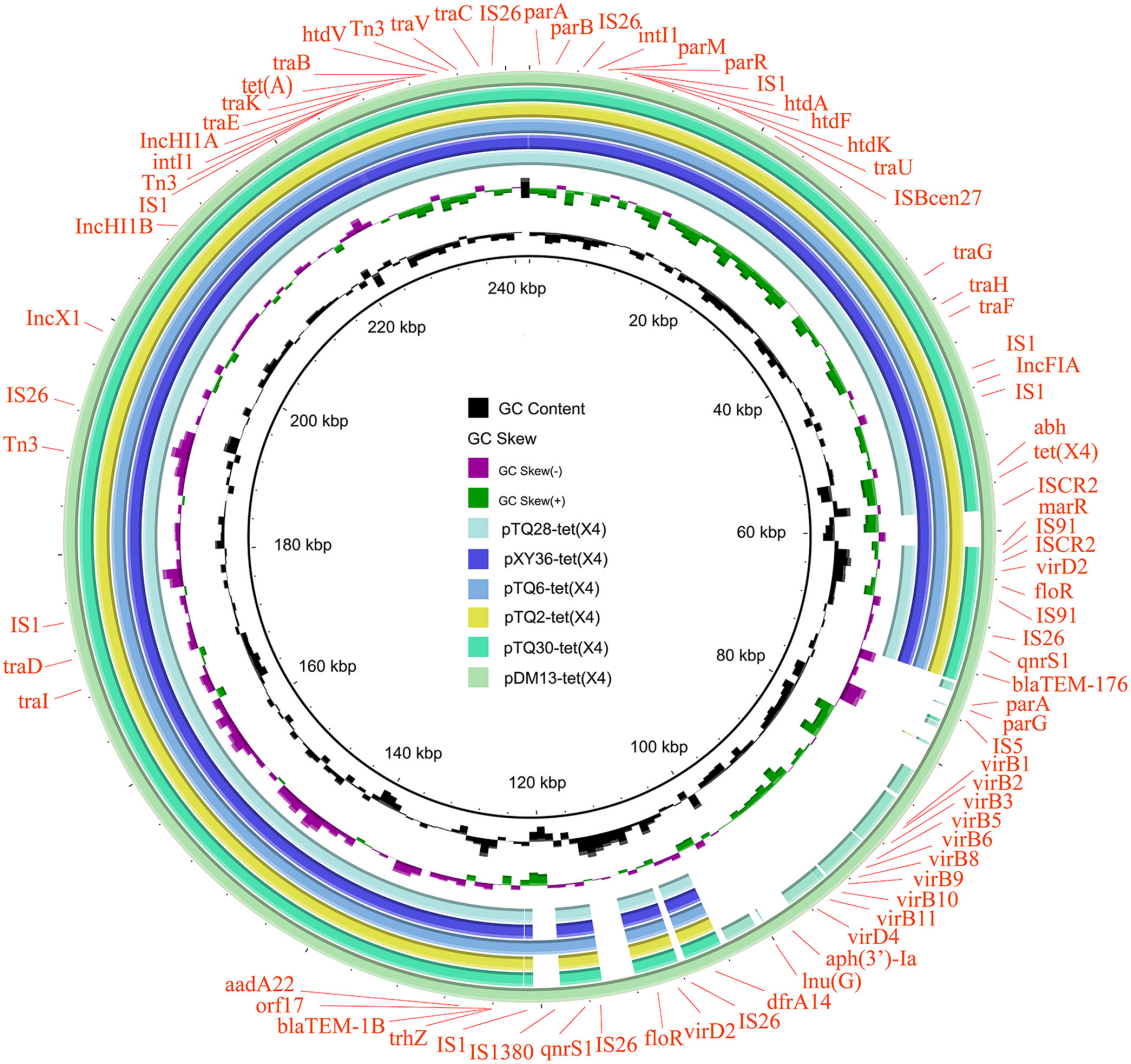
Comparative analysis of the IncFIA-IncHI1A-IncHI1B plasmids. Plasmids of different species are shown in circles without color. The replicons of the plasmids, insertion sequences, and resistance genes were marked with orange text. Plasmid size could be seen in Table 2.

Based on the results of the bacterial complete genome map, the genetic contexts of *tet*(X4) were analyzed and categorized into five main groups, as follows: group I had the most normal structure of IS*26-abh-tet*(X4)-IS*CR2-virD2*, which consisted of 9 strains in this study. There was a different upstream IS*CR2* of *tet*(X4) performing IS*CR2-abh-tet*(X4)-IS*CR2-virD2* in group II (*n* = 3) compared with that in group I. An analysis of group III (*n* = 1), namely, IS*CR2-abh-tet*(X4)-IS*CR2*, revealed that IS*CR2* was located upstream and downstream of *tet*(X4), whereas group IV (*n* = 2) had an upstream IS*1B*, forming the genetic structure of IS*1B-abh-tet*(X4)-IS*CR2-virD2.* Compared with group IV, there was no *virD2* downstream in group V (IS*1B-abh-tet*(X4)-IS*CR2*, *n* = 4). Besides, interestingly, pJZ21-*tet*(X4) had seven *tet*(X4) genes, which was consistent with the second-generation sequencing results depicting that the copy number of *tet*(X4) was approximately 10 times that of other drug-resistant genes. However, the number of *tet*(X4) genes was not proportional to the MICs (Fig. 3 and 4). Moreover, 68.42% of plasmids carrying *tet*(X4) and 100% of ICEs carrying *tet*(X6) could be transferred successfully with a conjugation frequency 10^−2^ to 10^−7^ and 10^−4^ to 10^−5^, respectively, and pTQ28-*tet*(X4) in M. morganii could horizontally transfer in E. coli strain EC600 with a conjugation frequency 10^−6^ (Table 2).

## DISCUSSION

Since the discovery of *tet*(X4), many studies have investigated the prevalence of tigecycline resistance in various regions of China, indicating different levels of prevalence and MDR and the risk of cloning transmission in different regions and sources (29–32). However, in the Sichuan Province, one of the largest pig farming provinces, studies on the prevalence of *tet*(X4)-positive bacteria are limited, and an analysis of their genetic environments is not sufficiently comprehensive. Therefore, we isolated *tet*(X4)/*tet*(X6)-positive *Enterobacterales* in pig samples from a pig slaughterhouse and 10 pig farms in Sichuan Province and analyzed the epidemic situation of *tet*(X4)-positive strains in this study. We isolated 35 *tet*(X4)/*tet*(X6)-positive strains and observed that Sichuan Province had a high prevalence rate (9.44%) of *tet*(X4)-positive bacteria in pig breeding, which was higher than that observed in previous studies. Because the tigecycline resistance strains were collected using resistant culture medium in this study, which perhaps provided a higher detection rate, the prevalence of *tet*(X4) observed from ordinary surveillance might have been underestimated. Therefore, continuous and specific monitoring of tigecycline resistance is important.

Additionally, this study demonstrated that except for E. coli, which was the dominant bacterium and a huge reservoir of the *tet*(X4) gene, there were six other species of bacteria carrying *tet*(X4) and exhibiting bacterial host diversity. Slaughterhouses had more types of bacteria than pig farms, and more people worked in a slaughterhouse than on farms, which causes a greater risk of drug-resistant bacteria for the workers in the slaughterhouse. There have been several clinical cases of different species of bacteria carrying *tet*(X4), such as K. pneumoniae and C. freundii (14, 16, 33). And M. morganii was first observed to carry tigecycline resistance gene *tet*(X4) in this study. The expansion of the host range of *tet*(X4) has promoted people to pay more attention to the potential threat of tigecycline resistance to human health. Besides, all *tet*(X4)/*tet*(X6)-positive strains exhibited high resistance to tigecycline (16 mg/L to 256 mg/L) and MDR. In a previous study, *tet*(X4) existed in animals, food, and the environments and spread horizontally among them, which posed a major threat to public health security (32, 34–37).

Furthermore, research has indicated that *tet*(X4) was observed to coexist with other antibiotic resistance genes (ARGs), such as *cfr*, *bla*_NDM-1_, and *mcr-1* (19, 38, 39). Furthermore, all *tet*(X4)-positive strains of this study coexisted with *floR*, of which most were still with *aadA*, *qnrS*, *dfrA*, *qacE*, *lnu*(F), *sul*, and *tet*(A), indicating that these ARGs may cotransfer with *tet*(X4). This cotransfer possibility poses a major challenge to the clinical use of antibiotics in an era where we rely primarily on antibiotics to treat diseases. The phylogenetic tree of 533 *tet*(X4)-positive E. coli strains exhibited that these strains arise from 8 sources and belong to 135 STs worldwide. The SNP differences among 533 *tet*(X4)-positive strains indicated that the *tet*(X4)-positive E. coli could clonally spread (SNPs, ≤5) and horizontally transfer among animals, humans, and the environment, which had similarities with previous studies (40). Thus, it is necessary to observe the transmission of tigecycline resistance genes. In addition, except for 35 tigecycline resistance strains carrying *tet*(X4) or *tet*(X6), the remaining 66 strains carrying tetracycline resistance genes *tet*(A), *tet*(J) and *tet*(M) were also resistant to tigecycline and 80.3% of these strains were *Proteus*. It has been found that K. pneumoniae and *E. hormaechei* carrying the *tet*(A) variant are resistant to tigecycline due to double frameshift mutation of the *tet*(A) gene (41, 42). And the *tet*(M) variant can make Streptococcus suis resistant to tigecycline (43). Previous studies have reported that P. mirabilis is inherently resistant to tetracycline antibiotics and that P. mirabilis strains carrying tigecycline resistance gene cluster *tmexCD3-toprJ3* have been detected from slaughterhouses (44–46).

Some studies have found that IS*CR2*, IS*1*, IS*26*, and a variety of conjugative and mobilizable plasmids of different incompatibility groups play an essential role in the acquisition of *tet*(X) genes from natural reservoirs and further dissemination among different bacterial pathogens (18, 47). In this study, we suggested that IS*CR2* was the main insertion sequence to mediate the transmission of *tet*(X4) among different plasmids. Plasmids, as important mobile genetic elements, play a crucial role in carrying multiple functional genes and transferring them across bacteria through conjugation (48). IncQ1 and IncX1 are reportedly the most widely distributed plasmids carrying the *tet*(X4) gene. Particularly, small IncQ1 plasmids can be transferred at a high frequency (10^−2^ to 10^−5^) in the presence of autobiographic helper plasmids (IncX4, IncI2, and IncFII). Additionally, only a few plasmids containing *tet*(X4) in E. coli isolates were single replicons, whereas most plasmids were multiple replicons, which may be derived through recombination among different plasmids, such as IncX1-IncN, IncX1-IncR, IncX1-IncFIA/B-IncY, IncX1-IncFIA/B-IncHI1A/B, and IncFIA/B-IncHI1A/B (3, 28, 32, 49). In this study, a structural analysis was carried out in 21 nonclonal strains after Nanopore sequencing. The results demonstrated that the IncX1 plasmid was the most prevalent plasmid. Interestingly, the length of IncX1 plasmid ranged from 31,271 bp to 73,084 bp, and it was discovered from different farms, slaughterhouses, and bacteria, suggesting a wide range of transmission. The similarity between pJZ18-*tet*(X4) and pYY76-1-2 from a sample of cattle in China was more than 99.9%, suggesting similarity in their origin. The 7 IncX1 types of plasmids had more than 99% similarity, suggesting that the plasmids mediate the horizontal transmission of *tet*(X4) in different bacteria. Studies have reported that IncX1 plasmids and their hybrid plasmids can cause epidemics in E. coli strains from pig farms in China and that *tet*(X4)-carrying IncX1 plasmids can spread among bacteria in humans and animals (28, 50). Furthermore, we elaborated on several other types of plasmids and are the first to report that *tet*(X4) was located on the heterozygous plasmid containing IncX3 in livestock E. coli and the IncA/C plasmid in livestock Proteus. These findings not only expand our understanding of the genetic environments of *tet*(X4) but also enable bacteria to better adapt and cope with different and complex environments when faced with selection pressure. In addition, since the discovery of *tet*(X6) in P. terrae subsp. *cibarius* in 2020, many reports have found that *tet*(X6) can coexist with the novel tigecycline resistance gene cluster *tnfxB3*-*tmexCD3*-*toprJ1b* and that *cfr* and *tet*(X6) can be transferred by ICE, which has many similarities with the results of this study (51–53). Finally, 68.42% of plasmids carrying *tet*(X4) and 100% of ICEs carrying *tet*(X6) in this study were transferred by conjugation experiments, indicating that plasmid-mediated and ICE-mediated horizontal transfer may occur in different pig farms and slaughterhouses since, of 10 pig farms, 2 had breeding pigs and 8 had fat pigs and parts of fat pigs were sent to the slaughterhouse.

In summary, in this study, we conducted in-depth research on the prevalence and genetic environments of the *tet*(X4) resistance gene in the pig slaughterhouse and farms in Sichuan Province and analyzed a total of 509 *tet*(X4)-positive E. coli strains observed in the 27,802 genomes of E. coli assembled from NCBI during 2002 to 2022 worldwide, providing a better understanding of the epidemiology and diversity of mobile genetic elements carrying *tet*(X4). We observed that the proportion of *tet*(X4)-positive strains was extremely high, and *tet*(X4) generally coexisted with other ARGs in different species of bacteria. Importantly, this report is the first one to demonstrate that *tet*(X4) was observed in M. morganii and that *tet*(X4) coexisted with *bla*_NDM-1_ in a strain of *K. quasipneumoniae*. Furthermore, the IncX1 plasmid was the most prevalent plasmid carrying the *tet*(X4) gene, and the insertion sequence IS*CR2* plays a significant role in transferring *tet*(X4) and in plasmid fusion. It also indicated that *tet*(X4) was located on an IncA/C plasmid in livestock Proteus and a heterozygous plasmid containing IncX3 in livestock E. coli. We performed an evolutionary phylogenetic analysis of 533 *tet*(X4)-positive E. coli strains worldwide and observed that these strains arise from 8 sources and belong to 135 STs. The results of this study expanded the host range and diversity of the plasmid of *tet*(X4), which may pose a serious threat to public health, and hence, more attention should be paid to monitoring tigecycline resistance and developing effective control measures. Additionally, we also should be concerned that tetracycline resistance may drive tigecycline resistance, and perhaps we need to reduce tetracycline use in pig farms.

## MATERIALS AND METHODS

### Sample collection and bacterial isolates.

In total, 300 fresh fecal samples were collected from 10 pig farms (30 for each farm), and 53 cecal samples were collected from a large-scale pig slaughterhouse in Sichuan Province, China, in 2021. One gram of fresh samples was cultured in 5 mL brain heart infusion (BHI) broth containing tigecycline (4 mg/L) and incubated at 37°C for 10 h in a shaking incubator at 180 rpm, to obtain tigecycline resistance cultures. Cultures were lined with the inoculating ring onto eosin methylene blue (EMB) agar plates containing tigecycline (4 mg/L) to obtain the tigecycline resistance Gram-negative bacterium using the three area marking method. Monoclones of different forms were selected for each plate, and the single colonies were stored in BHI broth with 25% glycerinum at −80°C. *tet*(X) resistance genes were determined by PCR and Sanger sequencing (3), and the *tet*(X)-positive isolates were further subjected to 16S rDNA sequencing for species identification.

### Antimicrobial susceptibility testing (AST).

Susceptibility to 17 antimicrobial agents, including gentamicin (GEN), florfenicol (FFC), polymyxin B (PMB), ciprofloxacin (CIP), trimethoprim-sulfamethoxazole (SXT), meropenem (MEM), cefoxitin (FOX), fosfomycin (FOS), aztreonam (ATM), doxycycline (DOX), norfloxacin (NOR), tetracycline, tigecycline, ceftazidime (CAZ), amoxicillin (AMC), ceftriaxone sodium (CRO), and amikacin (AMK), was determined using the Kirby-Bauer disk diffusion method. E. coli ATCC 25922 was used as the quality-control strain. All results were interpreted per the guidelines of the Clinical and Laboratory Standards Institute and the European Committee on Antimicrobial Susceptibility Testing. The MICs of tigecycline for the strains carrying *tet*(X) were determined using the broth dilution method (MIC breakpoints of tigecycline from the European Committee on Antimicrobial Susceptibility Testing are as follows: susceptible (S), ≤1 mg/L; 1 mg/L < intermediate (I) ≤ 4 mg/L; and resistant (R), >4 mg/L).

### Genome sequencing and bioinformatic analysis.

Total DNA of 101 tigecycline resistance strains was extracted using a TIANamp bacterial DNA kit (Tiangen, China) and was quantified by a NanoDrop 2000 instrument. Genomic DNA was sequenced using the Illumina NovaSeq 6000 platform with paired-end sequencing of 150 bp and assembled using SPAdes version 3.15.3 (54). We downloaded 27,802 E. coli genomes from the RefSeq database of NCBI and used the local blast program to build a library of these genome sequences. We used the *tet*(X4) gene as the query sequence to screen *tet*(X4)-positive E. coli. Antimicrobial resistance genes and multilocus sequence type (MLST) were determined using online tools (http://www.genomicepidemiology.org/). Prokka was used to annotate these genomes (55). The phylogenetic trees of positive strains of tigecycline resistance genes were constructed using Roary and FastTree based on single nucleotide polymorphisms (SNPs) of core genomes (56, 57). The resultant phylogeny was visualized and modified using iTol (https://itol.embl.de). A threshold of 5 SNPs among isolates is considered clonally related and likely to have an epidemiological link (58). MLST allelic profiles of E. coli were conducted using GrapeTree (59).

Nonclonal and representative strains (bacteria with large differences in drug resistance phenotype, drug resistance gene, and MICs) were selected for long-read Nanopore sequencing per the results of AST, phylogenetic analysis, and Illumina sequencing. The rapid barcoding kit RBK004 was used to construct DNA libraries, which were further sequenced in a MinION sequencer with a Flo-MIN106 flow cell. The genome sequences were completed with a hybrid *de novo* assembly strategy combining Illumina short-read and Nanopore MinION long-read data using Unicycler version 0.4.8 software (60). Plasmid replicons were determined using online tools (https://cge.cbs.dtu.dk/services/). Insertion sequences were discovered using ISfinder (https://www-is.biotoul.fr/index.php). The integrative and conjugative elements (ICEs) were detected by BLASTn analysis (http://blast.ncbi.nlm.nih.gov/Blast.cgi). The complete genome sequences were annotated automatically using RAST (http://rast.nmpdr.org/) and were modified manually. BRIG and Easyfig were used to display plasmid comparison maps (61, 62).

### Conjugation experiments.

A conjugation assay was conducted using the filter mating method, using rifampicin-resistant E. coli EC600 as the recipient, to examine the transferability of *tet*(X)-positive strains. The donor and recipient were mixed at a ratio of 1:4 in BHI, were cultured until the logarithmic growth period, and were further applied to sterilized 0.22-μm filters in Luria-Bertani agar plates, which were incubated at 37°C overnight. Transconjugants were selected on EMB agar plates containing 4 mg/L tigecycline and 400 mg/L rifampicin and further confirmed with PCR. Transfer frequencies were calculated as the ratio of the number of transconjugants to the total number of recipients.

### Data availability.

The sequences obtained in this paper have been deposited in the GenBank database under BioProject number PRJNA827787. Genome accession and accession numbers can be seen in Table 1 and 2.
